# Use of a novel clip-band device for difficult biliary cannulation due to papillary edema after precutting

**DOI:** 10.1055/a-2134-7448

**Published:** 2023-08-21

**Authors:** Koichiro Mandai, Tomoya Ogawa

**Affiliations:** Department of Gastroenterology, Kyoto Second Red Cross Hospital, Kyoto, Japan

Duodenoscopic clipping is often challenging because the duodenoscope elevator may disrupt the opening and rotation of the clip. However, the SureClip (Micro-Tech Co. Ltd., Nanjing, China), which provides enhanced rotational and re-opening performance, facilitates easier clipping while using a duodenoscope 1 . Recently, a novel clip-band device called the SureClip Traction Band (Micro-Tech Co. Ltd.) was developed and reported to be a useful traction device for endoscopic submucosal dissection 2 . The device consists of the SureClip and an elastic silicone band. Here, we describe the use of a novel clip-band device for difficult biliary cannulation due to significant edema of the major papilla after pre-cutting.

A 77-year-old woman with common bile duct stones was referred to our department. Endoscopic retrograde cholangiopancreatography (ERCP) was attempted; however, biliary cannulation was unsuccessful even after precutting with a needle knife. Three days later, a second ERCP was performed, which revealed significant papillary edema that hindered the visibility of the incision site ( Fig. 1 ). Therefore, a traction device was used to improve visibility. First, a metallic clip (SureClip Traction Band) was placed at the left edge of the incision site ( Fig. 2 a ). The elastic band was then grasped with another metallic clip (SureClip), pulled toward the left side of the papilla, and attached to the duodenal mucosa a few cm away from the papilla ( Fig. 2 b ). The same procedure was performed on the right edge, which resulted in improved visibility ( Fig. 3 ). Biliary cannulation was achieved using the double-guidewire technique with a double-lumen cannula (Uneven Double Lumen Cannula; Piolax Medical Devices, Kanagawa, Japan) 3 4 ( Fig. 4 ). Biliary drainage was performed using a 7 Fr double pigtail plastic stent ( Video 1 ).

**Fig. 1:**
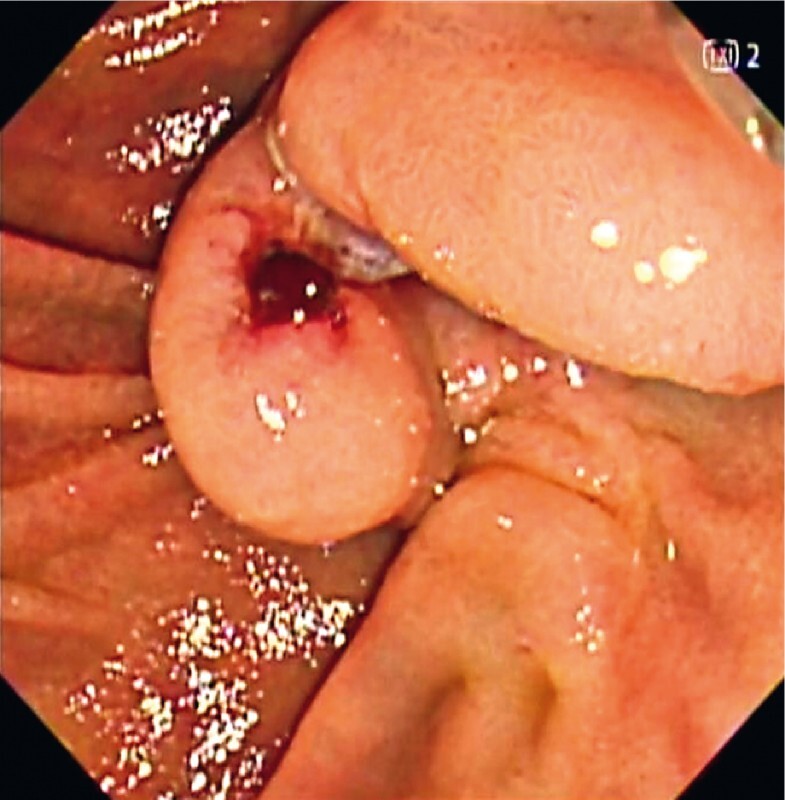
Endoscopic images showing significant edema in the papilla after precutting, which obscured the visibility of the incision site.

**Fig. 2 a:**
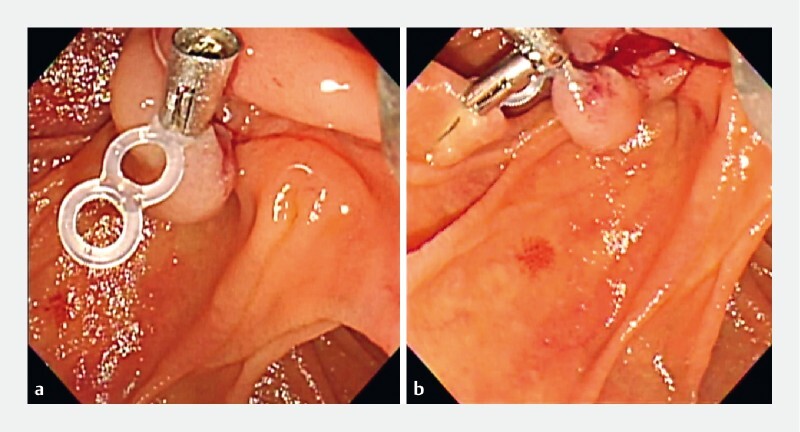
Endoscopic image showing a metallic clip with an elastic band placed at the left edge of the incision site. **b** Endoscopic image shows that the first clip with the elastic band is towed by the second clip attached to the duodenal mucosa a few centimeters away from the papilla.

**Fig. 3:**
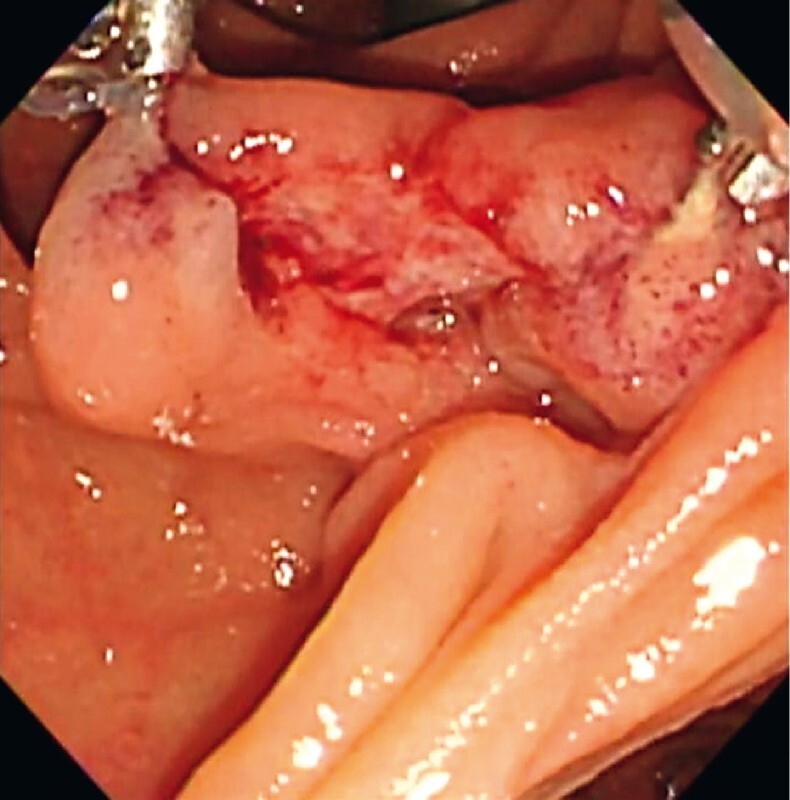
Endoscopic image showing improved visibility of the incision site after the traction of the incision edges.

**Fig. 4:**
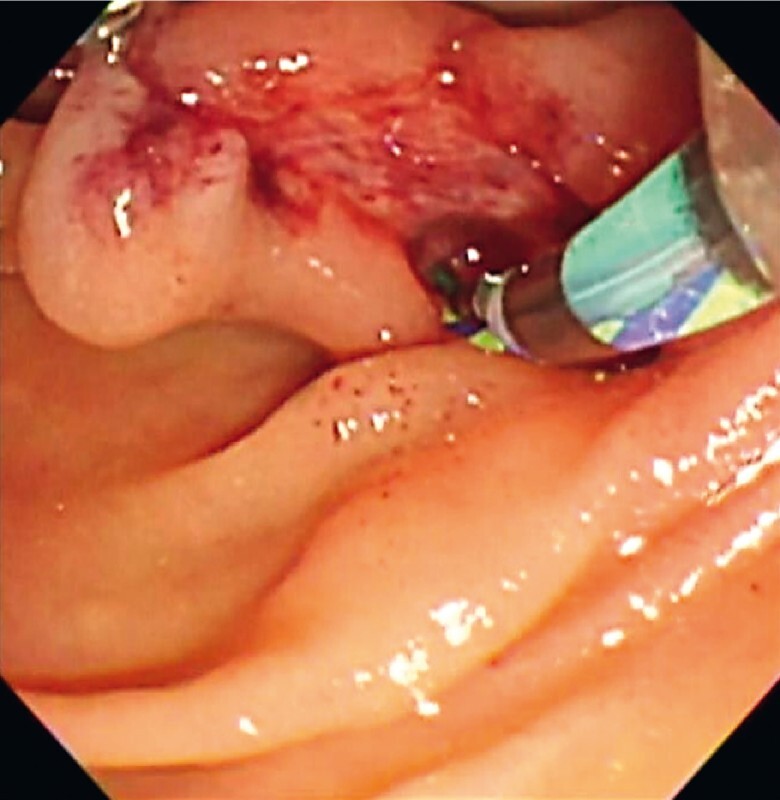
Endoscopic image showing a guidewire inserted into the bile duct using a double-lumen cannula.

**Video 1** Significant edema observed in the papilla after precutting, which obscured the visibility of the incision site. Visibility was enhanced using the novel clip-band device and successful biliary drainage was performed.


This case shows the use of a novel clip-band device to improve the visibility of the papilla.

Endoscopy_UCTN_Code_TTT_1AR_2AC

## Notes

Endoscopy E-Videos

https://eref.thieme.de/e-videos
*E-Videos* is an open access online section of the journal *Endoscopy* , reporting on interesting cases and new techniques in gastroenterological endoscopy. All papers include a high-quality video and are published with a Creative Commons CC-BY license. Endoscopy E-Videos qualify for HINARI discounts and waivers and eligibility is automatically checked during the submission process. We grant 100% waivers to articles whose corresponding authors are based in Group A countries and 50% waivers to those who are based in Group B countries as classified by Research4Life (see: https://www.research4life.org/access/eligibility/ ). This section has its own submission website at https://mc.manuscriptcentral.com/e-videos
